# The Influence of Shape Parameters on Unidirectional Drug Release from 3D Printed Implants and Prediction of Release from Implants with Individualized Shapes

**DOI:** 10.3390/pharmaceutics15041276

**Published:** 2023-04-19

**Authors:** Vanessa Domsta, Christin Hänsch, Stine Lenz, Ziwen Gao, Farnaz Matin-Mann, Verena Scheper, Thomas Lenarz, Anne Seidlitz

**Affiliations:** 1 Institute of Pharmacy, Biopharmaceutics and Pharmaceutical Technology, University of Greifswald, Felix-Hausdorff-Str. 3, 17489 Greifswald, Germany; 2 Department of Otorhinolaryngology, Head and Neck Surgery, Hannover Medical School, Stadtfelddamm 34, 30625 Hannover, Germany; 3 ENT Institute and Department of Otorhinolaryngology, Eye & ENT Hospital, Fudan University, Shanghai 200031, China; 4 NHC Key Laboratory of Hearing Medicine, Fudan University, Shanghai 200031, China; 5 State Key Laboratory of Medical Neurobiology and MOE Frontiers Center for Brain Science, Institutes of Biomedical Sciences, Fudan University, Shanghai 200032, China; 6 Cluster of Excellence “Hearing4all” EXC 1077/1, 30625 Hanover, Germany; 7 Institute of Pharmaceutics and Biopharmaceutics, Heinrich-Heine-University Düsseldorf, Universitätsstr. 1, 40225 Düsseldorf, Germany

**Keywords:** 3D printing, additive manufacturing, implants, drug release, Eudragit^®^, prediction, geometry, shape, individualization, frontal neo-ostium

## Abstract

The local treatment of diseases by drug-eluting implants is a promising tool to enable successful therapy under potentially reduced systemic side effects. Especially, the highly flexible manufacturing technique of 3D printing provides the opportunity for the individualization of implant shapes adapted to the patient-specific anatomy. It can be assumed that variations in shape can strongly affect the released amounts of drug per time. This influence was investigated by performing drug release studies with model implants of different dimensions. For this purpose, bilayered model implants in a simplified geometrical shape in form of bilayered hollow cylinders were developed. The drug-loaded abluminal part consisted of a suitable polymer ratio of Eudragit^®^ RS and RL, while the drug-free luminal part composed of polylactic acid served as a diffusion barrier. Implants with different heights and wall thicknesses were produced using an optimized 3D printing process, and drug release was determined in vitro. The area-to-volume ratio was identified as an important parameter influencing the fractional drug release from the implants. Based on the obtained results drug release from 3D printed implants with individual shapes exemplarily adapted to the frontal neo-ostial anatomy of three different patients was predicted and also tested in an independent set of experiments. The similarity of predicted and tested release profiles indicates the predictability of drug release from individualized implants for this particular drug-eluting system and could possibly facilitate the estimation of the performance of customized implants independent of individual in vitro testing of each implant geometry.

## 1. Introduction

Three-dimensional (3D) printing represents a manufacturing process with a high degree of freedom regarding the shape design of printed objects. Thus, this technique offers great potential in the growing field of individualized medicine, since dose adjustments, controlled drug release profiles and variable shapes are attracting increasing interest.

Nowadays, a broad spectrum of literature deals with 3D printing of drug-loaded products, such as films, tablets or capsules [1,2,3,4,5]. Different geometrical designs were developed within these categories to control the drug release mechanisms, for example by 3D printing of release-controlling shells, varying shell and infill designs, as well as hollow or multi-compartment systems [6,7,8,9,10]. The manufacturing of drug-eluting implants represents a special area of application for the use of 3D printing techniques, because for certain implantation sites, it may be beneficial or even necessary for the desired clinical outcome to adapt the implant shape to the anatomy of the individual patient. In the literature, multiple aspects such as different materials, the influence of additives or implant designs have been investigated to achieve 3D printed implants with diverse characteristics, for example, in terms of mechanical or drug release properties [1,11]. 3D printing of medical devices in shapes that fit the patient-specific anatomy is already performed for surgical training or as guidance tools, as well as for the manufacturing of patient-specific instruments or custom implants [12,13,14]. Most of these individualized products do not contain active ingredients, whereas the drug-eluting implants described in the literature are mostly based on simplified geometries or several standard sizes [11]. The number of medical devices with patient-specific shapes in combination with the incorporation of active pharmaceutical ingredients is limited [15,16,17], and until now, clinical trials on 3D printed drug-eluting medicinal products have not been performed to the authors’ knowledge. However, an individual healing trial has been reported for the treatment of a patient with external ear canal atresia or stenosis using a customized 3D printed implant containing ciprofloxacin and dexamethasone [15]. Consequently, the influence of the shape adaption to the patient’s anatomy on the drug release properties has not been extensively studied for customized drug-eluting implants so far. In consideration of the high variability of human anatomic structures, resulting in implants with highly variable shapes with different volumes and surface areas, an influence on the released amounts of drug is expectable and may also influence the therapeutical effects.

The importance of the shape of dosage forms as an influencing parameter on drug release is basically known since many studies on the release behavior of tablets with different shapes have already identified those phenomena [18,19,20,21,22,23]. Furthermore, 3D printing was used for the manufacturing of tablets with different external or internal shapes in combination with the investigation of their release behavior [24,25,26,27,28,29,30,31]. For a long-term drug release in the case of implants, non-dissolvable materials or materials with slow degradation characteristics are mainly used. Consequently, the drug release mechanism is often primarily based on diffusion processes and is not easily comparable to studies with soluble or easily erodible materials used for oral dosage forms. Moreover, the transferability of findings from simple geometries on complex anatomy-adapted shapes is still pending.

Since the anatomy of each patient is unique, the application sites for customized implants are manifold. In this study, the region of the frontal ostium of the frontal sinus was exemplarily chosen as a possible location for implants with patient-specific shapes due to its high variability [32]. The gold standard in surgical treatment for patients with chronic rhinosinusitis who require restoration of drainage and ventilation pathways is functional endoscopic sinus surgery. By the surgical removal of obstructive bone, tissue formations, polyps and scar tissue from the frontal ostium, a frontal neo-ostium is created to open the frontal sinus airway [32]. The postoperative care in these patients is substantial to prevent them from restenosis of the frontal sinus pathway by scar tissue or synechiae leading to revision surgeries. Postoperative drainage and wound healing could possibly be improved by the intraoperative manual insertion of a hollow implant using surgical forceps. The purpose of the implant is to mechanically prevent restenosis of the frontal neo-ostium and to release drugs into the surrounding tissues to reduce inflammation similar to the purpose of drug-eluting sinus stents [32,33,34]. Currently, there are two drug-eluting sinus implants on the market that are used intraoperatively after surgery for the treatment of patients with chronic rhinosinusitis: Propel™ sinus implant (Medtronic plc, Dublin, Ireland) and Relieva Stratus™ MicroFlow Spacer (Acclarent, Inc., Menlo Park, CA, USA) [35,36,37,38]. The use of these implants also implies some disadvantages as they only come in a few sizes and are not available in customized shapes, which have to be addressed for the improvement of postoperative care. 

The aim of this work was to study the influence of shape variations of model implants with simplified geometry on the drug release profile and determine possible consequences for the manufacturing of customized 3D printed implants. For this purpose, suitable materials and process parameters were identified for uniform 3D printing of bilayered hollow cylindrical model implants with different defined shape parameters and with unidirectional release of the model drug paracetamol to the external side over several days. The influence of different volumes, surface areas and area-to-volume ratios (A/V) of the drug-loaded matrix were compared by studying drug release from implants of different heights and wall thicknesses. Furthermore, the obtained data were used for the mathematical characterization of the release mechanism and for the determination of a predictive calculation of drug release for implants with patient-specific shapes, which were 3D printed according to the anatomical structures of three different patients. 

## 2. Materials and Methods

### 2.1. Materials

The water-soluble model drug paracetamol (water solubility according to manufacturer: 14 g/L at 20 °C), also known as acetaminophen, was purchased from Caesar & Loretz GmbH (Hilden, Germany). The used water-insoluble polymers Eudragit^®^ RS PO/ RS 100 and Eudragit^®^ RL PO/ RL 100 (ammonio methacrylate copolymer type A and B) were kindly donated by Evonik Industries AG (Essen, Germany). Triethyl citrate (TEC; Merck Schuchardt OHG, Hohenbrunn, Germany) was used as a plasticizer. Further used chemicals were methanol (VWR International S.A.S., Rosny-sous-Bois, France), sodium chloride (Caesar & Loretz GmbH, Hilden, Germany), potassium chloride (Carl Roth GmbH + Co. KG, Karlsruhe, Germany), calcium chloride monohydrate (Caesar & Loretz GmbH, Hilden, Germany or Carl Roth GmbH + Co. KG, Karlsruhe, Germany), 2-(N-morpholino)ethanesulfonic acid monohydrate (Carl Roth GmbH + Co. KG, Karlsruhe, Germany) and sodium hydroxide (Caesar & Loretz GmbH, Hilden, Germany). Additionally, a conventional 3D printable white filament with a diameter of 2.85 mm based on polylactic acid (PLA) was purchased from Formfutura BV (Nijmegen, The Netherlands).

### 2.2. Workflow 

In this study, bilayered implants with a simplified hollow cylindrical geometry, as well as implants with patient-specific shapes, were 3D printed from filaments prepared by hot-melt extrusion (HME) and characterized for their visual appearance, uniformity and drug release behavior. The release data of the hollow cylindrical implants were characterized by release kinetic models and were included in mathematical prediction calculations to enable the prediction of release profiles of implants with patient-specific shapes. 

**Figure 1:**
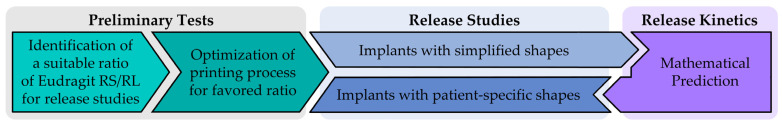
Schematic overview of the performed workflow.

However, preliminary tests had to be performed beforehand as visualized in Figure 1. These included the release testing of bilayered implants based on different ratios of Eudragit^®^ RS and RL to identify a suitable mixture for the following release studies, as well as procedures for the optimization of the printing process with the favored drug-releasing polymer ratio. The performed methods are described in the following sections. Those are structured to the general methods including the proceeding of manufacturing and characterization used for all experiments and followed by specific information on the preliminary tests, as well as on the implants with simplified hollow cylindric and patient-specific shapes.

### 2.3. General Methods

#### 2.3.1. Hot-Melt Extrusion of Drug-Loaded Filaments

Drug-loaded filaments were produced via HME as feedstock material for the 3D printing process. Initially, powder mixtures of the used polymers and the model drug paracetamol were prepared using a shaker mixer (Turbula^®^ T2F, Willy A. Bachofen AG, Muttenz, Switzerland) at 49 rpm for 10 min. In some compositions, the liquid plasticizer TEC was included. In these cases, the plasticizer was manually incorporated into the powder mixture before final mixing in the shaker mixer. Mixtures without TEC showed insufficient flowability using the finely powdered Eudragit^®^ RS/RL PO. Consequently, for those compositions, ground Eudragit^®^ granules were used instead. The granules were frozen at −80 °C before grinding in a tube mill (IKA^®^ Tube Mill 100 control, Staufen, Germany) at 25,000 rpm for a few seconds and sieving through a sieve with a mesh size of 500 µm. The specific compositions of the used mixtures are listed in Table 1.

The resulting powder mixtures were processed into filaments by HME using a co-rotating twin-screw extruder equipped with a flat-tray feeder, conveyor belt (ZE 12, ZD 9 FB and Conveyor, Three-Tec GmbH, Seon, Switzerland) and a water-cooled inlet zone (CF30 Cryo-compact circulator, JULABO GmbH, Seelbach, Germany). A round-shaped die with an inner diameter of 2.8 mm was used to produce filaments of an intended diameter of 2.85 mm, which would be ideal for the applied 3D printing process. Only filaments with determined diameters between 2.70 mm and 3.00 mm were used as feedstock material for the 3D printing process. Table 2 lists the main parameters of the extrusion process, which were adjusted to suitable conditions individually for each batch.

#### 2.3.2. 3D Printing Process and Implant Design

Model implants were printed from the drug-loaded filaments and drug-free PLA filaments using the fused deposition modeling (FDM) dual nozzle 3D printer Ultimaker 3 (Ultimaker B.V., Utrecht, The Netherlands; equipped with QR^TM^ extruder from Bondtech AB, Värnamo, Sweden). The main principle of the FDM 3D printing technique includes the feeding of a filament towards a heated nozzle and the layer-by-layer deposition of the molten material onto a build plate. The desired shape of the printed object is built up by defined pathways in x-, y- and z-directions by movements of the print head or build plate. 

The implant designs were constructed using the computer-aided design (CAD) software FreeCAD 0.18 (https://www.freecad.org, accessed on 26 October 2019) and converted into a G-code, readable by the 3D printer, using the slicing software Cura 4.8.0 (version 4.4 for in preliminary tests; Ultimaker B.V., Utrecht, The Netherlands). All implants were printed using nozzles with an inner diameter of 0.4 mm and blue painter’s tape on the build plate to improve the adhesion of the first printing layers. Conventional PLA filaments were used to transfer the force of the feeding and retraction mechanism onto the filaments produced by HME inside the bowden tubes of the printer, because the filaments were too short to reach the feeding mechanism and their resistance to the driving gears had not been tested.

**Figure 2:**
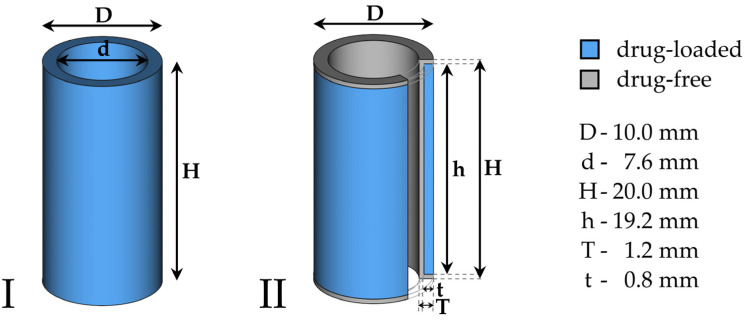
Schematic image (CAD) of monolayered (**I**) and bilayered (**II**) hollow cylindrical model implants with standard dimensions. D: outer diameter, d: inner diameter, H: total height, h: height of the drug-loaded part, T: total wall thickness, t: wall thickness of the drug-loaded part.

A hollow cylinder was selected as a model implant design with dimensions based on the anatomy of the human frontal neo-ostium as an exemplary option for an application site. For the determination of suitable printing parameters, such as the printing temperature, as listed in Table 2, a hollow cylinder with standard dimensions of 20 mm of height, an outer diameter of 10 mm and a wall thickness of 1.2 mm (Figure 2(I)) was printed from each drug-loaded filament composition. Subsequently, these printing parameters were transferred onto the dual-extruder printing process for bilayered model implants, which were printed with a mostly inert drug-free layer on the luminal side, as well as on the top and bottom of the implant, with a thickness of 0.4 mm (Figure 2(II)) using conventional PLA filaments. The drug-loaded part composed as previously determined was printed on the abluminal side of the implant only. For this purpose, the two separate extruders of the 3D printer were loaded with the drug-loaded and drug-free filaments. During the printing process, the printer switched the active nozzle in each layer to deposit the corresponding printing lines. This design was intended to achieve a unidirectional drug release exclusively from the abluminal surface of the implant, which would be in contact with the mucosa in vivo and additionally prevent the removal of the drug by the intended drainage of physiological fluid through the lumen of the implant.

#### 2.3.3. Visual Appearance

The appearance of the individual implants was visually investigated after the printing process to evaluate the effect of parameter changes during the development and optimization process. Representative images were taken photographically, as well as using a reflected light microscope (Zeiss Stemi 2000-C with Zeiss CL 1500 ECO, AxioCam ICc 1 and AxioVision software, all Carl Zeiss AG, Oberkochen, Germany).

#### 2.3.4. Uniformity of Mass, Dimensions, Drug Dose and Content

The mass and the dimension of at least eight samples were determined to evaluate the uniformity of the printed implants. The height, as well as the inner and outer diameter, were measured using a digital caliper under the exclusion of the slightly expanded first printing layers. Each dimension was determined at four different measuring points for each cylindrical sample.

The relative drug content of the paracetamol-containing part of the implants was determined in triplicate for every printed design. Therefore, the implants were shaken in 20 mL methanol for 24 h in a horizontal shaker (KL-2, Edmund Bühler GmbH, Bodelshausen, Germany) at 300 rpm and room temperature. The drug-containing Eudragit^®^ part was dissolved in the methanol during this period, whereas the PLA part remained. After centrifugation (Centrifuge 5702 R, Eppendorf SE, Hamburg, Germany; 4000 rpm, 15 min) and appropriate dilution, the absorption of the solution was measured spectrophotometrically (Cary^®^ 50, Agilent Technologies, Inc., Santa Clara, USA; 10 mm quartz cuvette) at a wavelength of 248 nm. Afterwards, the remaining PLA part was weighed after evaporation of residual solvent to determine the mass of the drug-loaded Eudragit^®^ part as the difference between the total implant mass and the mass of the undissolved remainder. The relative drug content was calculated by the measured paracetamol amount related to the mass of the drug-loaded Eudragit^®^ part of the implant.

#### 2.3.5. Drug Release Studies

**Figure 3:**
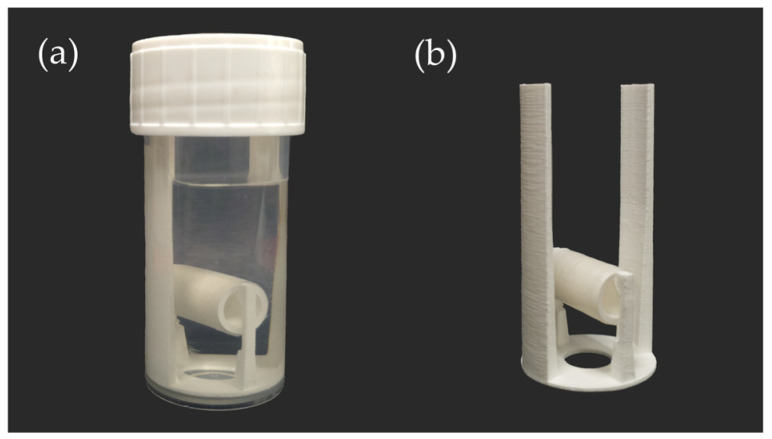
Photographs of the setup of drug release studies. (**a**) Model implant fixed on the mount inside the release medium. (**b**) Model implant on the mount during transfer into fresh medium.

Drug release studies were performed by incubating the implants in 20 mL of release medium in a horizontal shaker (IKA^®^ KS 3000i control, IKA^®^ Werke & CO. KG, Staufen, Germany) at 100 rpm and 37 °C. Simulated nasal fluid pH 6.5 was used for the preliminary tests with the same composition, as already described in the literature at the same or related pH values [39,40,41,42,43,44,45]. For all further release tests, a buffering agent was added to a medium of a quite similar composition (bSNF; buffered simulated nasal fluid), containing 7.45 g/L sodium chloride, 1.39 g/L potassium chloride, 0.32 g/L calcium chloride monohydrate and 10 mM 2-(N-morpholino)ethanesulfonic acid monohydrate. The pH was adjusted to 6.5 using sodium hydroxide. Three implant samples of quite similar total mass were included in the release studies to reduce the impact of printing variations and focus on the influence of the implant shape. During the release studies, the implants were fixed on a 3D printed mount (Figure 3) to ensure complete contact of the drug-containing part of the implant with the release medium and to enable a simple transfer into fresh preheated media at predefined sampling time points. Adhering release media on the surface of the mount was carefully removed during the transfer using low-lint cellulose tissues to minimize carryover effects. The released amounts of paracetamol in the media samples were analyzed spectrophotometrically (Cary^®^ 50, Agilent Technologies, Inc., Santa Clara, CA, USA; 10 mm quartz cuvette) after suitable dilution at a wavelength of 243 nm. The percentage fraction of released drug were related to the theoretical total drug dose of the implants as 10% of the individual mass of the drug-loaded part. The release studies were terminated at the plateau phase at the end of the release profile when only minimal UV absorbances were detectable in the samples of the previous 24 h sampling period, except for release from implants based on RSRL(100:0), which was terminated after 21 days. The plateau phase with negligible changes in released drug levels is not depicted for all cases in the result section for clarity.

### 2.4. Preliminary Tests

Preliminary tests were performed on the different filament compositions and the optimization of the printing process before performing experiments with implants of simplified hollow cylindrical shapes or with patient-specific shapes.

#### 2.4.1. Polymer Ratio of Eudragit^®^ RS and RL

Bilayered model implants were printed from filaments of mixtures of Eudragit^®^ RS and RL in different ratios or the pure polymers, respectively. Those were tested for their drug release behavior to identify a suitable polymer ratio for the intended experiments with implants of different shapes. A drug release period of 5 to 7 days was intended for the implants of standard dimensions in order to identify possible differences in long-term drug release and allow a high number of different shapes to be tested within the study period.

#### 2.4.2. Optimization of Printing Process

After the estimation of a suitable mixture of 80 parts Eudragit^®^ RS and 20 parts Eudragit^®^ RL on the basis of the results of the drug release from preliminary tests, three different concentrations of TEC were added to this polymer ratio as a plasticizer (plasticizer-polymer ratio 2:98, 5:95 and 10:90). The intention was to improve the flowability of the molten material for accurate printing results and to improve the flexibility of the filaments for a reduced risk of filament fractures due to the applied feeding forces during the printing process.

Since the geometrical parameters of the implants, such as area and volume, were calculated from the CAD models, the density of the printing lines has to be as homogeneous as possible for all designs to transfer the calculated values to the printed implants. To achieve this, the resolution of the implant designs exported as a stereolithography (STL) file was increased, and the relevant printing parameters, for example, the printing temperature, speed, line width, overlapping or extrusion factor, were adjusted. The investigated printing parameters for the favored composition “RSRL(80:20)_5T” and PLA used for the experiments on the bilayered implants with simplified hollow cylindrical and patient-specific shapes are listed in Table 3. The density of the drug-loaded Eudragit^®^ part was calculated by the mass soluble in methanol in relation to the theoretical volume of the part calculated from the CAD model. 

### 2.5. Implants with Simplified Shapes

In order to analyze the effects of shape variability on the drug release behavior, hollow cylindrical implants were printed from the paracetamol-containing filaments “RSRL(80:20)_5T” and PLA filaments with different defined shape parameters.

#### 2.5.1. Implant Designs of Simplified Shapes

Three series of implants in different dimensions were designed by varying the height and wall thickness of the drug-loaded part of the bilayered hollow cylindrical implants resulting in different volumes, surface areas and, consequently, different area-to-volume ratios regarding the drug-loaded part (Table 4). 

In the design A series, the height was varied with constant wall thickness resulting in a constant ratio of area-to-volume. The design B series had varied wall thicknesses with a constant height and consequently constant surface area. For the design C series, varied wall thicknesses were combined with adjusted height to achieve similar volumes. The implant design with standard dimensions was part of every design series (A1, B2 and C1).

#### 2.5.2. Estimation of Drug Release Kinetics and Release Prediction 

The mechanisms of drug release kinetics for different pharmaceutical dosage forms, for example matrix or reservoir types, can be described by various mathematical models [46,47,48]. These models can be helpful in understanding the release mechanisms of the tested dosage forms and deriving important information for further optimization or predictions. The mathematical model which fits the best with the obtained release data was estimated by the highest coefficient of determination *R*^2^ of linearization plots representative of releases profiles following the equation of zero order, first order, Higuchi, Weibull or Korsmeyer-Peppas (Table 5). The mean data points of release studies from the hollow cylindric implants were included in the calculations up to 80% of fractional release.

The mentioned mathematical, empirical or semi-empirical kinetic models enable the characterization of several drug release mechanisms, including matrix-based systems [46,47,48]. The Korsmeyer-Peppas equation describes the drug release behavior from controlled polymeric devices [53,54] and fitted well with the obtained release data. Therefore, it was used for the prediction of drug release. For dosage forms with filled cylindrical geometry the Korsmeyer-Peppas model is based on the one-dimensional radial drug release under sink conditions and the second Fickian law. The constant *k_K_* includes structural and geometrical characteristics of the dosage form. The diffusional exponent *n* indicates the drug transport mechanism by Fickian diffusion, anomalous transport or zero-order release, whose value is also dependent on the geometry [54].

The involved parameter of the Korsmeyer-Peppas equation can be determined by slope and intercept from the linearization plot of logarithmized percentage fraction of released drug against logarithmized time. Additionally, mathematical relations between the release constant *k_K_* and geometrical parameters of tested hollow cylindrical implants were identified to predict the drug release from implants with patient-specific shapes. In addition to the fraction of released drug, the predicted absolute released drug amounts were calculated based on the theoretical drug load of 10%, the volume of the drug-loaded part in the CAD model and the density of printing lines for the specific implant design. The predicted release profiles were compared to the experimental release data and difference factor *f*_1_

(1) $$f_{1}=\left[\frac{\sum_{t=1}^{n}\left|R_{t\;}-T_{t}\right|}{\sum_{t=1}^{n}R_{t}}\right]\;\cdot \;100$$

as well as similarity factor *f*_2_

(2) $$f_{2}=50\;\cdot \;\log+\log\;\left[\frac{100}{\sqrt{1+\frac{\sum_{t=1}^{n}{\left(R_{t}-T_{t}\right)}^{2}}{n}}}\right]$$

were calculated to quantify their degree of congruence, where *n* is the number of compared time points and *R_t_* or *T_t_* are the release values of predicted and tested profiles [55,56]. Time points were included in the calculation according to the experimental sampling intervals below 80% of cumulative drug release. Shorter intervals at the beginning of the release studies were used to ensure the detection and comparison of potential burst release rates.

### 2.6. Implants with Patient-Specific Shapes

Implants exemplarily adapted to the shape of bony edges of frontal neo-ostium structures were designed, 3D printed and characterized to estimate the effects of customized shapes on the release behavior and compare the experimental release data with the predicted release profiles.

#### 2.6.1. Acquisition of Anatomical Shape of Human Frontal Neo-Ostium 

Cone beam computer tomography (CBCT) images of human sinuses were reconstructed and exported as Digital Imaging and Communications in Medicine (DICOM) data. The responsible ethics committee approved the use of the patient’s data (approval code: 1897-2013).

The structures of the frontal neo-ostia were manually segmented from the DICOM-data using 3D Slicer^TM^ version 4.11 (https://www.slicer.org, accessed on 30 September 2020), as previously described [32]. A surface smoothing effect with the dimensionless parameter of 0.5 was applied during the processing of the implant surface from segments and the result was exported as an STL file. Three frontal neo-ostia structures from three different patients were selected for further experiments. These have different sizes and also include overhanging structures, which may pose a challenge regarding the 3D printing process. 

#### 2.6.2. Modeling of Patient-Specific Implants

**Figure 4:**
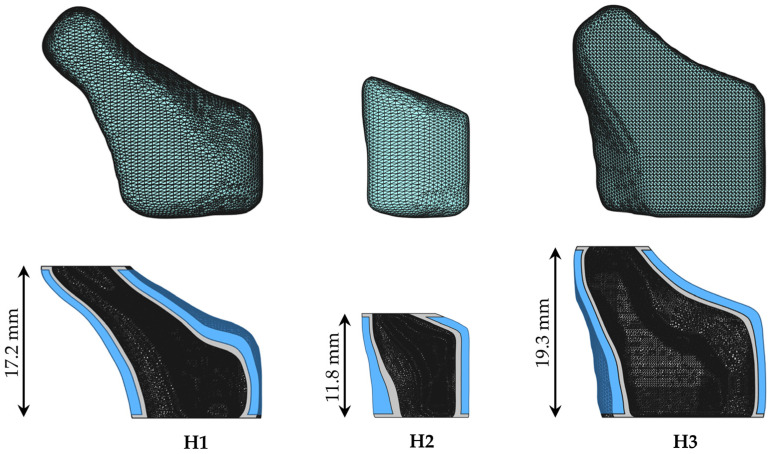
Exported STL files of individual human frontal neo-ostial structures after segmentation from CBCT images (**upper row**) and modeled bilayered hollow implants with patient-specific shapes (**lower row**); grey: drug-free inlay, blue: drug-loaded part.

The exported STL files (Figure 4, upper row) of individual human frontal neo-ostium structures were hollowed using the software Autodesk Meshmixer 3.5 (Autodesk, Inc., San Rafael, CA, USA). Shells with different wall thicknesses were created in steps of 0.4 mm, corresponding to the nozzle diameter of the 3D printer and, thus, the resulting thickness of individual printing lines. Further processing was performed equally to the simple geometries of the cylindrical bilayered implant by creating an upper and lower opening for drainage and applying a 0.4 mm thick inner, upper and lower inert layer using FreeCAD. A wall thickness of 0.8 mm was selected for the drug-loaded part for all three patient-specific implants (Figure 4, lower row), similar to the hollow cylindrical implants of standard dimensions. Furthermore, it was expected that the parameter of wall thickness would intuitively be kept constant by operators who want to print customized implants, as this parameter also affects the mechanical properties of the implants. 

## 3. Results

### 3.1. Preliminary Tests

#### 3.1.1. Drug Release from Different Polymer Mixtures

**Figure 5:**
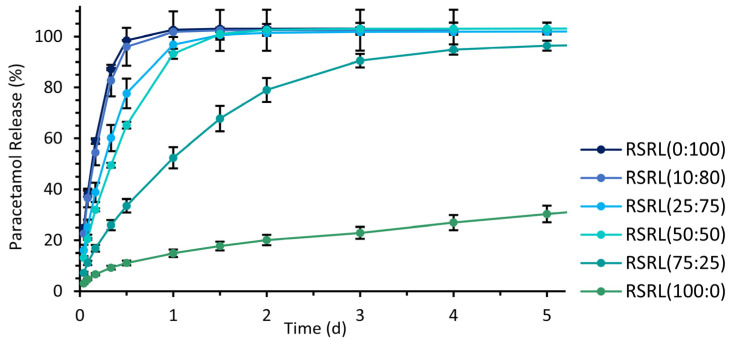
Paracetamol release profiles of bilayered hollow cylindrical implants in standard dimensions based on different Eudragit^®^ RL and RS ratios in SNF; mean ± SD, *n* = 3.

Bilayered hollow cylindrical implants with an inner PLA layer and an outer drug-loaded part based on Eudragit^®^ RS and RL in different ratios were 3D printable in standard dimensions. The release profiles of these implants demonstrated rapid drug release within 1 day from polymer mixtures with a high proportion of Eudragit^®^ RL and decelerated drug release up to several days by increasing proportions of Eudragit^®^ RS (Figure 5). The drug release period of 5 to 7 days targeted for further investigations was not achieved by the tested ratios of Eudragit^®^ RS and RL but could be estimated between a ratio of 75:25 and 100:0 regarding these release profiles. Thus, a ratio of 80:20 was used for further optimization of the printing process. 

#### 3.1.2. Optimized Printing Process

Adjustments of the composition, as well as the model processing and printing parameters, were performed to optimize the quality of the 3D printed implants. A homogeneous drug-loaded part was intended to ensure the correctness of implant volumes and areas calculated from the CAD models.

The addition of TEC as a plasticizer to the Eudragit^®^ RS/RL base in a plasticizer-to-polymer ratio of 5:95 (RSRL(80:20)_5T) was identified as a suitable composition for homogeneous printing results. Those filaments were neither too brittle nor too flexible to enable appropriate feeding to the heated nozzle and a homogeneous deposition of the molten polymer strands. 

**Figure 6:**
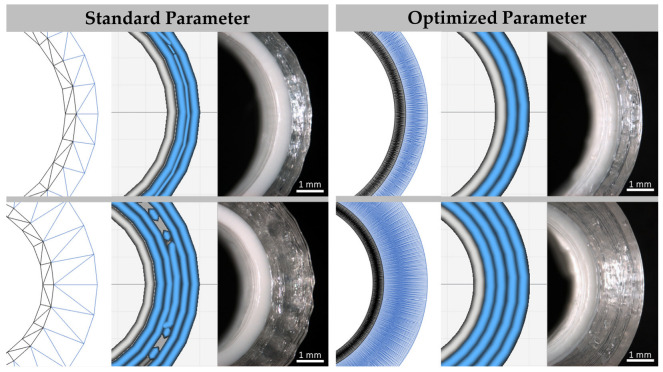
STL format, printing pathways and microscopic images of bilayered hollow cylindrical implants (**left** to **right**) prepared with standard and optimized parameters, exemplarily shown for implants with a wall thickness of 0.8 mm (**upper row**) and 1.6 mm (**lower row**) of the drug-loaded part; gray/white: drug-free inlay (PLA), blue/transparent: drug-loaded part (RSRL(80:20)_5T).

The printing process with standard parameters resulted in inhomogeneous drug-loaded parts of the implants caused by interrupted deposition of the printing lines and lines with an only partial thickness (Figure 6, left). The curvatures in the STL format seem smoothed by the usage of a higher resolution for the triangulated surface of the cylindrical model. Combined with optimized printing parameters, especially such as the line width, overlapping and extrusion factors, printing pathways with continuous lines of homogenous thickness were obtained for different wall thicknesses, each as a multiple of the nozzle diameter of 0.4 mm (Figure 6, right). The level of homogeneity of the drug-loaded implant part was quantified by the determination of its density (Table 6). The density of printing lines of hollow cylindrical implants was relatively uniform with a value of 1.18 ± 0.06 mg/mm³ for all designs. The obtained densities for implants with patient-specific shapes with mean values between 1.12 mg/mm³ and 1.20 mg/mm³ were in the same range, although smaller holes and thinner walls could occur in the printing pathway. This is caused by the creation of shells by the inset of the three-dimensional triangular mesh by 0.8 mm, which is not identical to a wall thickness of 0.8 mm in the printing direction of the x/y-section plane.

### 3.2. Implants with Simplified Shape

#### 3.2.1. Characterization of Implants with Simplified Shape

**Figure 7:**
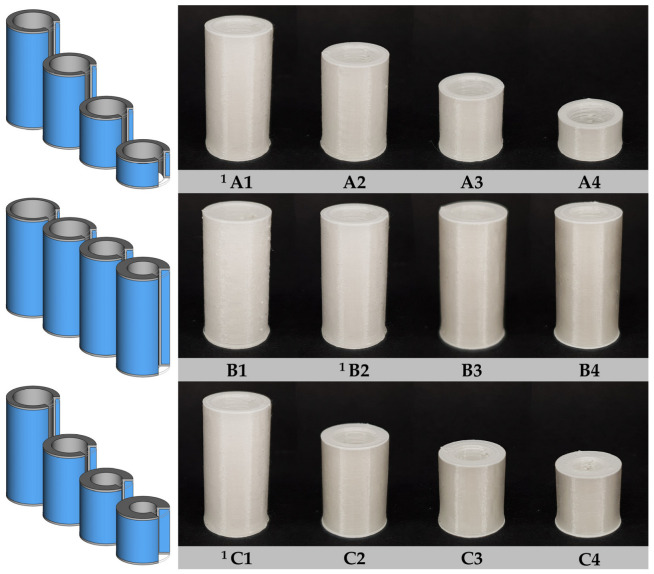
Schematic and photographic images of simplified hollow cylindrical implants with same area-to-volume ratio (A1–A4), area (B1–B4) or volume (C1–C4); grey/white: drug-free inlay (PLA), blue/transparent: drug-loaded part (RSRL(80:20)_5T); ^1^ Identical implant of standard dimensions (S).

All designs of implants with defined shapes of different heights and wall thicknesses were successfully 3D printed. The visual appearance is uniform with regular deposited transparent printing lines of “RSRL(80:20)_5T” and white printing lines of PLA (Figure 7). No defects in the implants such as voids were observed during visual inspection. The first few printing layers are a little broadened due to the adherence to the build plate. Implants of design B1 with only one drug-free and one drug-loaded printing line in the cross-section had some more irregularities in the printing lines compared to all other designs caused by the limited adhesion on previously printed lines and more frequent nozzle changes.

The printed hollow cylindrical implants were uniform within their design regarding their mass, dimensions, relative drug content of the drug-containing part and the total drug dose of the implants (Table 7). Variability regarding the relative drug content of the drug-containing part may arise from variable drug distribution within the drug-loaded matrix already affected by the drug distribution in the extruded filaments. Variability in the total drug dose is additionally influenced by the uniformity of the printing process regarding the amounts of extruded drug-loaded material. The highest deviations in the relative drug content and total drug dose were detected for implants of the design type B1 with single drug-free and drug-loaded lines. The determined geometrical dimensions fitted the targeted values of the designs. However, the mean inner diameter was about 0.5–0.8 mm smaller than the targeted value. 

#### 3.2.2. Drug Release from Implants with Simplified Shapes

The transfer of implants on the 3D printed mounts into fresh media enabled easy handling during release studies without mechanical forces acting on the implants themselves. Neither changes in the appearance of the implants and printing layers nor extensive swelling were visually recognized during the whole release period. 

**Figure 8:**
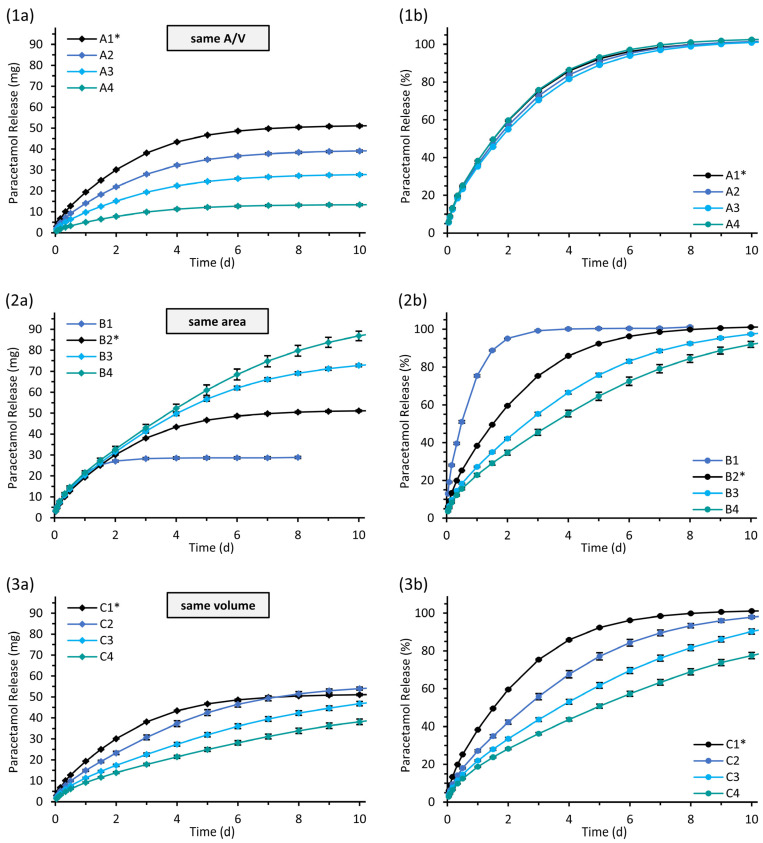
Paracetamol release profiles of bilayered hollow cylindrical implants of different designs with same area-to-volume ratio (**1**), area (**2**) or volume (**3**) of the drug-loaded part in bSNF; mean ± SD, *n* = 3. The diagrams on the left (**a**) show the cumulative amount of the released drug mass and the diagrams on the right (**b**) as the proportion of the expected total amount of 10% (*w/w*) paracetamol related to the drug-loaded part of the individual implant. * Identical implants of standard dimensions (S).

The release profiles of paracetamol from the hollow cylindrical implants are presented in Figure 8. The relative release rates from implants with the same area-to-volume ratio were similar, but the released amount of drug per time, as well as the total released drug dose, differed in dependence on the implant size (Figure 8(1a,1b)). Implants with the same surface area in contact with the test medium released similar drug amounts per time in the initial phase (Figure 8(2a,2b)). The period of time until completion of release was different for those implants, increasing with a higher wall thickness. Implants with the same volume are suggested to release equivalent total drug doses over the total release time. The release profiles show this tendency, but absolute, as well as fractional, drug release rates differ (Figure 8(3a,3b)).

#### 3.2.3. Drug Release Kinetics and Release Prediction 

The calculated values of *R*^2^ were at least 0.97 for the linearization plots of all kinetic models and implant designs (Table 8). The Korsmeyer-Peppas equation fitted the best (*R*^2^ > 0.999) with the drug release data obtained in our study for the hollow cylindrical implants. Thus, this kinetic equation was used for the mathematical prediction of drug release for the implants with varying patient-specific shapes.

The values of the diffusional exponent *n* and the logarithm of the kinetic constant *log*(*k*) of the Korsmeyer-Peppas equation were identified by the slope and intercept of the linear regression of the logarithmical original equation:

(3) $$log\left(Q_{t}\right)=n\cdot t+log\left(k\right)$$

**Figure 9:**
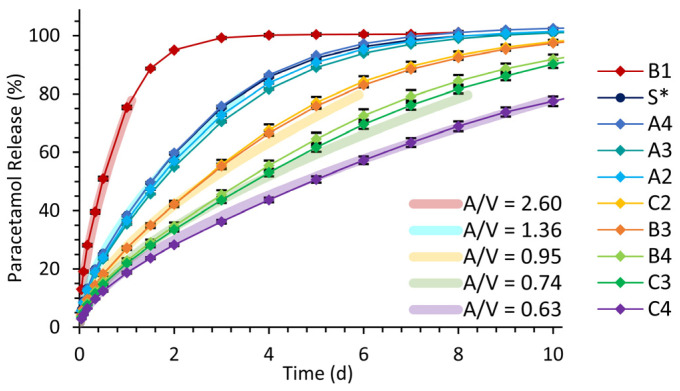
Release profiles of experimental data (thin lines, mean ± SD, *n* = 3) of performed release studies with hollow cylindrical implants and predictive curves (thick lines), depending on values of area-to-volume ratios (*A*/*V*) based on the Korsmeyer-Peppas kinetic equation. * Identical implants of standard dimensions (S).

The values of slope (representing *n*) were similar within each design, since this value represents the release mechanism. Consequently, further calculations were performed using the mean n value of all tested implant designs. Furthermore, similar values of intercepts (representing *log*(*k*)) could be recognized for implants with the same area-to-volume ratio. The following relation of *log*(*k*) and the area-to-volume ratio *A*/*V* was identified as

(4) $$log\left(k\right)=10^{a}\cdot {\left(A/V\right)}^{m}$$

with values of 0.2251 and 0.1650 for *m* and *a*, respectively, derived by the experimental data using a double-logarithmic linear regression of *log*(*k*) and *A*/*V*. Predictive release profiles dependent on the known area-to-volume ratio were calculated up to a release level of 80% by applying the identified constant values and known area-to-volume ratios to the original kinetic equation of Korsmeyer-Peppas (Table 5). The release profiles of all tested hollow cylindrical implants are illustrated in Figure 9 compared to the predicted release profiles for equivalent area-to-volume ratios. The predictive release profiles match well with the data points of the performed release studies.

### 3.3. Implants with Patient-Specific Shape

#### 3.3.1. Characterization of Implants with Patient-Specific Shape

**Figure 10:**
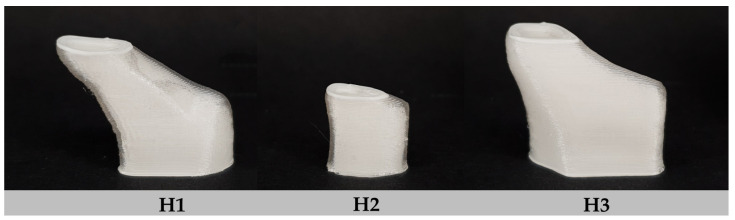
Photographic images of bilayered implants with patient-specific shapes according to their frontal neo-ostial anatomy; white: drug-free inlay (PLA), transparent: drug-loaded part (RSRL(80:20)_5T).

Photographic images of the successfully 3D printed implants, according to the anatomical frontal neo-ostium structures of three different patients, are presented in Figure 10. The deposition of printing layers was uniform. The complex surfaces appeared to be smooth, especially the larger and even areas, but irregularities of printing lines were also rarely detected in overhanging structures and sharper curvatures.

The quality of the printing results for these complex-shaped implants seemed to be as reproducible, as in the case of the hollow cylindrical implants, since the range of deviations regarding the mass, as well as total drug dose and relative drug content, were similar (Table 9).

#### 3.3.2. Drug Release from Implants with Patient-Specific Shape

**Figure 11:**
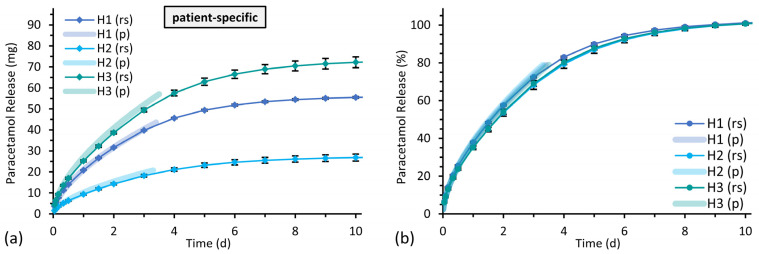
Experimental paracetamol release profiles (rs, mean ± SD, *n* = 3) of bilayered implants with different patient-specific shapes compared to prediction curves (p) for the equivalent area-to-volume ratios in bSNF. The diagram on the left (**a**) shows the cumulative amount of the released drug mass and the diagram on the right (**b**) as the proportion of the expected total amount of 10% (*w/w*) paracetamol of the drug-loaded part of the individual implant.

The drug release profiles from implants with patient-specific shapes demonstrated similar relative drug release rates but different released amounts of drug per time and total released drug doses. This release behavior is comparable to the release from cylindrical implants with constant wall thicknesses and the same area-to-volume ratios. The predictive curves for individual implant shapes were near the data points of the performed release studies (Figure 11). Difference factors *f*_1_ of 1.7%, 7.6% and 4.6% and similarity factors *f*_2_ of 96.0%, 73.1% and 84.1% for patient-specific implants H1, H2 and H3, respectively, indicated the equivalence of the release profiles from experimental and predicted data according to the FDA guidance, although the method is designed to compare experimental release data from different formulations of immediate release solid oral dosage forms and a larger sample size is required by the guidance [55].

## 4. Discussion

### 4.1. Eudragit^®^ RS/RL as Material for 3D Printed Implants

The non-soluble polymers Eudragit^®^ RS and RL are widely used in the pharmaceutical development of controlled oral dosage forms and are well applicable in manufacturing processes using HME or 3D printing [57,58,59]. In addition to tablets, capsules, discs and patches, implants based on at least one of these polymers have also been produced by 3D printing [57]. The hollow cylindrical implants of Eudragit^®^ RS, 3D printed by Kempin et al. [60], released only small amounts of the incorporated drug within several weeks. Consequently, this material could be suitable for implantable devices with an intended long drug-eluting period over months. In our preliminary tests, the cylindrical model implants based on Eudragit^®^ RS released about 30% of the model drug paracetamol within only 5 days. This indicates a long-term drug release over some weeks but is far less compared to the mentioned findings of Kempin et al. This discrepancy indicates a high dependency on drug release profiles on several factors such as geometrical shape, as well as the characteristics, of the specific drug-polymer system.

Controlling the drug release period was easily achieved by the use of mixtures of Eudragit^®^ RS and RL in different ratios, as demonstrated in the preliminary tests. This was beneficial for our further studies to adjust the duration of experiments within the intended period but would be also beneficial for the manufacturing of implants with different intended therapeutical times of action. Higher amounts of Eudragit^®^ RL increased the release rates, whereas Eudragit^®^ RS prolonged the release period. Similar dependencies of the drug release rates on the ratio of Eudragit^®^ RS and RL have been previously described in the literature for HME extrudates or 3D printed tablets by direct extrusion process [61,62].

According to the manufacturer’s technical information, including results of safety studies for oral, dermal, subcutaneous and intraperitoneal administration, Eudragit^®^ RS and RL have a very low to negligible toxicity [63]. Consequently, the specific risk of the processed implant out of this material has to be further estimated but can be presumed to be low. The tested formulations in our studies were 3D printable in good quality at temperatures between 140 °C and 155 °C. Paracetamol seems to be a suitable model drug, since FDM based 3D printing has been performed several times using this drug [1,64,65]. Furthermore, degradation of the model drug is not expected at the finally applied processing temperatures of 150 °C, as the initial onset of degradation of paracetamol has been reported in the literature at temperatures above 150 °C from thermogravimetric analyses [66,67,68,69,70], and the determined relative drug content was in the range of the theoretical drug load. The performed processing temperature is in the lower section of the temperature range described in the literature from 150 °C to 215 °C for FDM 3D printed dosage forms based on Eudragit^®^ RS or RL [57]. The optimal printing temperature is dependent on the specific printer and the influence of further excipients. Added plasticizers can reduce the needed processing temperature by decreasing the melt viscosity, but even incorporated drugs can have plasticizing effects [71,72]. In this study, the printability of Eudragit^®^ RS/RL filaments was improved by the usage of TEC as a plasticizer, but too-high amounts of TEC amounts in a plasticizer-polymer ratio of 10:90 resulted in filaments with strongly varying diameters and were consequently excluded from the printing process. The filaments containing TEC in a plasticizer-to-polymer ratio of 5:95 showed improved printability compared to the non-plasticized filaments of all tested polymer ratios regarding the improved rheological characteristic of the melt and decreased brittleness during the filament feeding process. Although the flexibility of the filament was increased by the plasticizer, the 3D printed monolayered implants were rigid, as well as the bilayered ones, in combination with the PLA inlay. The biodegradable polymer PLA was used as a conventional filament in technical grade with conformity for food contact, but the raw material is available in pharmaceutical grade as well. Furthermore, the processing of PLA via HME and FDM 3D printing for the manufacturing of drug-eluting implants was already demonstrated several times [11]. The internal PLA layer could comply with two main functions. Firstly, the drug-free barrier facilitates unidirectional drug transport to the surrounding tissue and reduces the removal of the drug by mucus drainage through the implant lumen. Secondly, mechanical stabilization of the implant shape is achieved, which is also relevant in vivo after the implantation. A shape deformation, including contraction and elongation, was observed in other preliminary tests for the monolayered hollow cylindrical implants based on Eudragit^®^ RS/RL when the implants were incubated at 37 °C surrounded by air or tissue-simulating agarose gel. These deformations were prevented by the PLA internal layer. Nevertheless, the rigid character of the bilayered implants does not comply with the optimal mechanical properties for the application to the frontal neo-ostium. For this specific application site, an implant should ideally be flexible to be compressed during insertion and returns to its original shape after positioning to provide mechanical stability and prevent re-occlusion of the neo-ostium. Flexible materials such as thermoplastic polyurethane (TPU) or ethylene vinyl acetate (EVA) have been processed via FDM 3D printing to drug-eluting systems in the field of catheters or gynecological devices [73,74,75,76] and could possibly meet the requirements of individualized implants for the frontal neo-ostium in the future. Thus, the exemplarily used patient-specific shapes acted as model geometries due to their high anatomical variability without the aim of a further application using the materials Eudragit^®^ RS/RL and PLA. Nevertheless, these rigid materials could be beneficial for other application sites, for example, in bony structures or orthopedic fixations, due to their good processability by 3D printing.

### 4.2. Optimization of the Printing Process for Homogeneous Implants

The geometrical characteristics of implants with simplified or patient-specific shapes were often calculated from the parameters of the CAD or STL models. However, the printing pathways and layerwise deposition of the extruded material during 3D printing affect the actual resulting values of surface area and volume. Thus, interpretations based on those calculations could be inaccurate if the printing process is not adapted to the requirements for this simplification. The actual surface area of 3D printed objects is enlarged compared to the modeled geometries by the radial end of each printing layer on the outer contour. Following the idealized assumption where the end of each layer is defined by a semicircle instead of a straight end, the area would be enlarged by more than 50%. Since the drug-eluting contour of the tested model implants was always orientated in the z-direction the factor of the enlarged surface would be similar for all and consequently negligible. Nevertheless, smoother contours would occur at areas parallel to the printing plate, and differences in the calculated surface area need to be considered if drugs could release from those areas. Optimal printing parameters are required to ensure a homogeneous filling of the modeled volume with drug-loaded material by the deposition of the round-shaped material strands. The degree of overlay of the strands needs to meet the requirements of the rheological properties of the individual compositions to achieve gapless connections. Little cavities could still arise in areas with sharp curvatures or wall thicknesses unequal to a multiple nozzle diameter. The filling of the resulting cavities in dimensions below the nozzle diameter can be performed by the use of smaller extrusion amounts in these areas but is limited by the imprecise deposition of very small material amounts using this printing technique. The tested hollow cylindrical implants were optimized regarding the homogeneous deposition of the printing lines and resulted in similar values of the determined density of printing lines. The density of patient-specific implants printed with identical printing parameters differed minimally caused by little cavities between the printing pathways as a result of their complex shape. Cavities in drug-eluting dosage forms could always affect the drug release by simplified penetration of the release media. For 3D printed oral dosage forms, an intended modification of drug release profiles was achieved by different infill densities or channels, whereas the drug release was significantly accelerated by larger cavities but only minorly by smaller cavities [18,28,77]. Regarding the minimal size and number of cavities in the implants with patient-specific shapes, their influence on the drug release behavior seems to be negligible. Alternatively, the volume of a 3D printed object could be calculated by the amount of extruded material during the printing process specified in the printing G-code. However, the calculated amounts may still differ from the actual extruded amounts, since this is highly dependent on the diameter of the fed filament, which may fluctuate within a certain range due to the manufacturing by HME [78].

The optimization of printing parameters for the tested material resulted in 3D printed implants with homogenously deposited printing lines in each layer, resulting in a smooth surface and gapless matrices. This enabled the geometrical characterization of drug-eluting parts based on the simplified CAD or STL model data. The 3D printed hollow cylindrical implants comply well with the intended dimensions with the exception of the inner diameter. Those were smaller than the targeted values due to shrinkage during the solidification of the molten material, a typical phenomenon in FDM 3D printing, especially regarding printed holes [79,80,81,82]. The observed small deviations in mass and total drug dose for each shape indicate a uniform and well-reproducible 3D printing process. Most of these deviations were probably caused by diameter fluctuations of the filament, but design B1 seems to be a special case with higher deviations. Printing only a single drug-loaded line and a single PLA line was especially challenging for this printing technique due to frequent material changes and limited adherence to previously printed lines. This resulted in partly irregular deposited printing lines and consequently higher variations in the drug-loaded part. Thus, two printing lines or more are advisable for the 3D printing of homogenous implants, if this is possible for the intended application site. 

### 4.3. Drug Release Studies

At the moment, there is no standard method defined for drug release testing of implants. Suitable methods could imply the use of the compendial or modified flowthrough cell, as well as dialysis or incubating methods in static or agitated bottles [83,84,85]. Taking the intended application site into account, the release medium should simulate the in vivo conditions of these areas regarding parameters such as osmolarity, pH, buffer capacity, flow or temperature but also maintain drug stability and sink conditions [84,85]. 

In this study, drug release testing was performed by incubation in agitated containers with implant transfer into fresh media as the sampling procedure due to its simple and reproducible setup. The paranasal sinuses are a complex construct of connected air-filled areas to the nasal cavity. Since the passage of secretion is circular in the sinuses and is drained to the nasal cavity [86], the characteristics of nasal fluid were considered for the choice of release media composition, even though drug release should occur into the tissue that is in direct contact with the dosage form. The amounts of electrolytes were related to the simulated nasal fluid described in the literature, which has already been used in multiple release tests for intranasal dosage forms [40,41,42,43,44] and is comparable to the composition of nasal fluid in vivo [87]. Furthermore, the pH and temperature values of the release medium were adapted to physiological conditions. Nasal mucus is slightly acidic [88,89,90,91]. No significant differences in pH value were found for patients with chronic rhinosinusitis, but pH values were lower after endoscopic sinus surgery [88]. The temperature increases with the depths of the airways [92,93] and is possibly close to the body temperature at the mucosal contact areas of the frontal neo-ostium. The simulation of the complex physiological balance between mucus secretion, transport and clearance [94,95,96] was not adequately feasible in the used simplified release method. Consequently, a reduced volume of 20 mL was chosen to ensure the complete contact of different implant shapes to the release medium. This volume was approximately three times the saturation volume of the tested sparingly soluble model drug paracetamol in the case of the implant with the highest drug dose to maintain unaffected drug release rates by sink conditions. Since the medium was replaced frequently, maintaining sink conditions was unproblematic using this media volume.

Strategies for an accelerated drug release study may be performed for long-acting dosage forms by alteration of method parameters such as temperature, pH value, level of agitation, solvent composition or the application of surfactants [83,84]. Those short-term release studies can be useful for quality control in the development or manufacturing phase, and they can provide predictive information on the long-term release behavior if a correlation exists to the accelerated release studies as described in the literature for the release from polymeric dosage forms [97,98,99]. Since the release period of several days was chosen by a suitable polymer ratio of the tested compositions in this study, these findings represent a demonstrative model for the predictability of release rates for individualized implants from simplified geometries. Furthermore, a correlation to long-term release studies for alternative polymer ratios might be further investigated, and the obtained findings may already provide useful information for implants with a release duration of several days.

### 4.4. Drug Release from Implants with Simplified Shapes

The adaption of the outer surface to the anatomical structures of the individual patient is of primary importance within the customization process of an implant. Moreover, the application of further changes in the shape design, for example, in volume or wall thickness of the implant, is conceivable to comply with the intended requirements of the mechanical properties or total drug dose. All this could affect the release of drug molecules due to the resulting smaller or greater distances and contact areas to the acceptor compartment. 

The influence of those parameters could be identified in the release profiles of implants with simplified hollow cylindrical shapes designed for the unidirectional drug release from the abluminal surface. Tested implants with similar volumes enabled the release of similar total drug doses, but the drug was released at different rates, increasing with larger surface areas and smaller wall thicknesses of the tested implants. Consequently, the applied drug amounts per day, as well as the duration of therapy, would differ. Similar absolute drug amounts per time were released from implants with similar surface areas in the initial release phase until the drug depot of the implants was depleted. Thus, the release rate of drug molecules per area on the contact phase of the implant and medium seems to be similar for the tested drug-polymer system, independent of the wall thickness. This could be beneficial in vivo if the contact areas are intended to be treated with similar drug amounts per day. In contrast, differences in surface areas should be evaluated critically in the cases of implants that are intended to enable the transport of the drug through the contact layer behind it or in deeper areas. Due to the similar absolute release rates from similar surface areas, the total drug dose and the duration of therapy are dependent on the implant volume and can possibly be controlled by the adaption of the wall thickness. The area-to-volume ratio was identified as the leading influence parameter on the relative drug release rates, since all release profiles of implants with the same ratio were congruent. This relation was shown for implant series A, including four implants with the same ratio, and underlined for further ratio values for implant designs B3 and C2 or B4 and C3 with the same ratios, respectively. The predominance influence of the area-to-volume ratio on the drug release was similarly described in the literature for tablets with different sizes or shapes manufactured by compression or 3D printing [19,20,24,26,31,100]. 

The following mechanisms influencing the drug release may occur within the tested composition based on Eudragit^®^ RS/RL: (1) water penetration into polymeric structures, (2) slightly polymer swelling, (3) release of dissolved drug molecules by diffusion-based pathways and (4) forming of pores due to dissolved drug and plasticizer. The release profiles of hollow cylindrical implants were well described by the Korsmeyer-Peppas equation. The interpretation of the release mechanism by the exponent *n* obtained by the Korsmeyer-Peppas equation is described for thin film, cylinders and spheres. Exemplarily, the limiting value of 0.45 for the release exponent *n* representing one-dimensional Fickian diffusion was defined for the first 60% of the drug release of filled cylinders with aspect ratios smaller than 0.2 [54]. The tested hollow cylindrical implants with unidirectional drug release do not comply with these conditions regarding their geometry and dimension. Consequently, a valid differentiation of the release mechanism from the obtained data is not easily feasible due to the geometrical dependency of exponent *n*. In the literature, the mass transport of different drugs from Eudragit^®^ RS or RL based delivery systems is described as mainly controlled by diffusion [72,101,102], which is probably the primary release mechanism for the tested implants. Due to the similarity of the calculated release exponents of the different tested shapes, the establishment of a mathematical prediction for drug release from the individualized implants based on the Korsmeyer-Peppas equation was applicable using the first 80% of drug release.

However, all stated findings on the drug release kinetics represent only the drug release mechanism for this specific drug-eluting system with the model drug paracetamol incorporated in a polymer mixture based on Eudragit^®^ RS and RL. Different types of drugs, drug loads and changes in the polymer matrix, including the amounts of plasticizer or further additives, might affect the drug release rates due to their different physicochemical properties. Ayyoubi et al. stated a more significant impact of composition than geometry on drug release for their tested 3D printed mini tablets [103]. Consequently, the release behavior has to be investigated always for the specific composition of the intended application. Drugs such as steroids, antibiotics, antiseptics, anti-inflammatory or antiproliferative agents could be beneficial for implantation in the presented application site after sinus surgery [34].

### 4.5. Predictability of Drug Release from Implants with Patient-Specific Shapes

The 3D printing technique provides great flexibility for the manufacturing of dosage forms of variable shapes. Thus, the characterization of release profiles and development of prediction tools were already performed using this technique [24,25,27,29,104]. Different sizes and network structures of the infill pattern were investigated to control and predict the drug release from tablets and implants [25,27,29,104], as well as different tablet geometries, such as cubes, a hollow cylinder, a cylinder or a pyramid [24]. Those prediction models seem to be promising tools to improve individualized drug therapy due to the mostly good fitting of predicted and experimental release profiles. This approach may also be a necessary prerequisite for the regulatory approval of shape-individualized dosage forms. 

The investigation of a suitable prediction method for drug release from long-acting implants with patient-specific shapes is especially important due to the high complexity of the customized shapes adapted to the individual patients’ anatomy. The results of this study demonstrated good prediction of release behavior for individualized implants with patient-specific shapes from data of in vitro release studies of implants with defined shapes of simplified geometry. The obtained release data from simplified implants with different surface areas, volumes and area-to-volume ratios were included in predictive calculations of patient-specific implants, exemplary produced and tested with the same wall thickness and, thus, similar area-to-volume ratios in three different shapes according to the individual neo-ostium. Further tests on patient-specific implants with different levels of area-to-volume ratios should be performed to confirm the findings of our study. Furthermore, extreme area-to-volume ratios should be tested to estimate the limits of the prediction, since this parameter was identified as highly influencing the drug release from the tested implants. It has to be pointed out that the wall thickness of the individual implant has to be kept constant using this approach. However, this is expected to be acceptable, since the mechanical stability of the implants is also an important factor. 

The extensive characterization, including drug release studies, of a sufficient number of implants with a customized shape would not comply with the idea of on-demand manufacturing of implants via 3D printing for improved individualized therapy. Thus, the opportunity for mathematical prediction of drug release for the individual patient from release data collection of an adequate wide range of different simplified test geometries, including physiological extrema, would be very beneficial. Depending on the application site and health status of the patient, different drug release rates, durations or total amounts of drug applications might be necessary for successful therapy. Knowing the anatomical shape and progress of the disease of the specific patient, the drug release from the individualized implant could possibly be estimated using mathematical equations, and optimization could possibly be performed by adapting the implant surface area or volume within the anatomical conditions. Certainly, for the application of 3D printing in the described ideal manner, the relationship between the area-to-volume ratio and drug release, as well as the predictability of release profiles, need to be investigated for further drugs, drug loads, polymers and implant shapes. Nevertheless, the obtained results demonstrate a great opportunity for implant characterization relevant to individually shaped implants but independent of the individual testing of the release behavior of each implant design.

## 5. Conclusions

An optimized 3D printing process was successfully developed for the manufacturing of bilayered hollow cylindrical implants containing the model drug paracetamol incorporated into a polymer matrix of Eudragit^®^ RS and RL, as well as a luminal diffusion barrier composed of PLA. Implants with different defined shape parameters were printed by the variation of the surface area and volume of the drug-loaded part and characterized in drug release studies. The ratio between area and volume was identified as the predominant influencing parameter for the release behavior. Moreover, a prediction of drug release based on the kinetic model of Korsmeyer-Peppas was implemented by the obtained data. The predicted release profiles, calculated dependent on the area-to-volume ratio of the implant, were similar to the release profiles of independently tested implants with three different patient-specific shapes, adapted to the anatomical structures of human frontal neo-ostium. Thus, the 3D printing technique is a promising tool for the manufacturing of individualized implants. Furthermore, the in vitro testing of the customized implants might be replaced by prediction models based on experiments with simplified geometries in the future.

## Figures and Tables

**Table 1:** Compositions of the powder mixtures for hot-melt extrusion (HME) of filaments; TEC: triethyl citrate.

Batch Name	Eudragit^®^ RS (% *w*/*w*)	Eudragit^®^ RL (% *w/w*)	TEC (% *w*/*w*)	Paracetamol (% *w*/*w*)	Eudragit^®^ RS:RL Ratio	Plasticizer: Polymer Ratio
RSRL(0:100)	0	90	-	10	0:100	-
RSRL(10:80)	10	80	-	10	10:80	-
RSRL(25:75)	22.5	67.5	-	10	25:75	-
RSRL(50:50)	45	45	-	10	50:50	-
RSRL(75:25)	67.5	22.5	-	10	75:25	-
RSRL(100:0)	90	0	-	10	100:0	-
RSRL(80:20)_2T	70.56	17.64	1.8	10	80:20	2:98
^1^ RSRL(80:20)_5T	68.4	17.1	4.5	10	80:20	5:95
RSRL(80:20)_10T	64.8	16.2	9	10	80:20	10:90

^1^ Composition used for implants with different hollow cylindrical and patient-specific shapes.

**Table 2:** Parameters of the HME process and printing temperatures.

Batch Name	Extruder Temperature (°C)			Screw Speed (rpm)	Feeding Rate (%)	Printing Temperature (°C)
	Zone 1	Zone 2	Zone 3–4			
RSRL(0:100)	120	130	140	15	1.5	155
RSRL(10:80)	110	140	150	30	2.0	155
RSRL(25:75)	100	120	140	20	1.8	155
RSRL(50:50)	100	120	130	20	1.8	155
RSRL(75:25)	90	105	115	20	1.7	155
RSRL(100:0)	90	110	115	20	1.7	155
RSRL(80:20)_2T	110	120	140	20	1.8	140
^1^ RSRL(80:20)_5T	80	110–115	115–120	20	2.7	150
RSRL(80:20)_10T	75	90	90	5	0.9	^2^ -

^1^ Composition used for implants with different hollow cylindrical and patient-specific shapes. ^2^ Excluded from printing tests due to high diameter variations of filaments.

**Table 3:** Main printing parameters of the 3D printing process of implants with hollow cylindrical and patient-specific shapes from paracetamol-containing filaments of the composition “RSRL(80:20)_5T” and polylactic acid (PLA).

Printing Parameter	RSRL(80:20)_5T	PLA
Printing Temperature (°C)	150	200
Print Speed (mm/s)	15	20–30
Layer Height (mm)	0.1	0.1
Initial Layer Height (mm)	-	0.3
Skin Overlap Percentage (%)	0	0
Line Width (mm)	0.4	0.4
Extrusion Factor “Flow” (%)	110	110
Outer Wall Line Width (mm)	0.385	0.385
Extrusion Factor “Outer Wall Flow” (%)	114	114
Build Plate Temperature (°C)	60	60
Build Plate Adhesion Type	Skirt	Skirt
Build Plate Adhesion	-	Blue Tape
Z Seam Position (Simplified Shape)	Sharpest Corner	Sharpest Corner
Z Seam Position (Patient-Specific Shape)	^1^ Right	^1^ Right

^1^ Patient-specific implants were placed on the build plate with the upper opening facing backward on the y-axis.

**Table 4:** Dimensions of the drug-loaded part (RSRL(80:20)_5T) of hollow cylindrical implant designs with same area-to-volume ratio (A), area (B) or volume (C). The calculated areas and volumes are related to the drug-loaded part of the implants. The calculated area describes only the abluminal surface of this part.

Design	Height (mm)	Wall Thickness (mm)	Outer Diameter (mm)	Calculated Area (mm²)	Calculated Volume (mm³)	Area/Volume Ratio (mm^−1^)
^1^ A 1	19.2	0.8	10	603.19	443.94	1.36
A 2	14.4	0.8	10	452.39	332.96	1.36
A 3	9.6	0.8	10	301.59	221.97	1.36
A 4	4.8	0.8	10	150.80	110.99	1.36
B 1	19.2	0.4	10	603.19	231.62	2.60
^1^ B 2	19.2	0.8	10	603.19	443.94	1.36
B 3	19.2	1.2	10	603.19	636.96	0.95
B 4	19.2	1.6	10	603.19	810.68	0.74
^1^ C 1	19.2	0.8	10	603.19	443.94	1.36
C 2	13.4	1.2	10	420.97	444.55	0.95
C 3	10.5	1.6	10	329.87	443.34	0.74
C 4	8.8	2.0	10	276.46	442.34	0.63

^1^ Identical implants of standard dimensions (S).

**Table 5:** Mathematical model equations for release kinetics and their linearization plots (y ↔ x).

Kinetic Model	Kinetic Equation	Linearization Plot		
Zero Order	$Q_{t}=k_{0\;}\cdot \;t$	%Q_t_	↔	t
First Order	$Q_{t}=1\;-\;{e\;}^{-k_{1\;}\;\cdot t}$	ln(100 − %Q_t_)	↔	t
Higuchi [46,49,50]	$Q_{t}=k_{H\;}\cdot \;\sqrt{t}$	%Q_t_	↔	$\sqrt{t}$
Weibull [51,52]	$Q_{t}=1\;-{e\;}^{\left[\frac{-{(t\;-\;T)}^{\beta }}{\alpha }\right]}$	log(−ln(1 − Q_t_))	↔	log(t)
Korsmeyer-Peppas [53,54]	$Q_{t}=k_{K}\;\cdot \;t^{n}$	log(%Q_t_)	↔	log(t)

Q_t_: Fraction of drug released at time t; %Q_t_ percentage fraction of drug released at time t; β: shape parameter; α: scale parameter; T: lag time; n: diffusional exponent; k_0_, k_1_, k_H_ and k_K_: release constants.

**Table 6:** Density of printing lines of the drug-loaded implant part; mean ± SD, *n* = 6.

Implant Design	^1^ All	^2^ S	A2	A3	A4	B1	B3	B4	C2	C3	C4	H1	H2	H3
Density (mg/mm³)	1.18 ± 0.06	1.16 ± 0.04	1.18 ± 0.03	1.21 ± 0.04	1.18 ± 0.04	1.26 ± 0.07	1.16 ± 0.02	1.16 ± 0.05	1.23 ± 0.01	1.18 ± 0.02	1.10 ± 0.02	1.13 ± 0.02	1.20 ± 0.07	1.14 ± 0.04

^1^ Mean ± SD of all tested hollow cylindrical implant samples (H1-3 excluded). ^2^ Identical implants of standard dimensions (also named A1, B2 and C1).

**Table 7:** Characteristics of 3D printed hollow cylindrical implants regarding their mass and dimension [targeted dimensions in square brackets] (mean ± RSD, *n* = 8), as well as the total drug dose and relative drug content (mean ± RSD, *n* = 3). The relative drug content defines the mass of paracetamol related to the mass of the drug-loaded part of the implant (RSRL(80:20)_5T).

Implant Design	^1^ S	A2	A3	A4	B1	B3	B4	C2	C3	C4
Mass (mg)	821.4 ± 3.3%	643.0 ± 3.8%	442.8 ± 2.4%	241.3 ± 1.9%	615.7 ± 2.5%	1031.4 ± 1.1%	1213.5 ± 3.2%	762.4 ± 1.4%	700.4 ± 2.6%	647.5 ± 1.4%
Outer Diameter (mm)	9.90 ± 0.49% [10.0]	9.94 ± 0.43% [10.0]	9.93 ± 0.23% [10.0]	9.92 ± 0.26% [10.0]	9.99 ± 0.70% [10.0]	9.86 ± 0.20% [10.0]	9.90 ± 0.47% [10.0]	9.96 ± 0.14% [10.0]	9.93 ± 0.32% [10.0]	9.89 ± 0.17% [10.0]
Inner Diameter (mm)	7.09 ± 0.80% [7.6]	7.11 ± 0.87% [7.6]	6.95 ± 0.66% [7.6]	7.02 ± 0.75% [7.6]	7.92 ± 0.59% [8.4]	6.21 ± 0.86% [6.8]	5.41 ± 1.40% [6.0]	6.01 ± 1.10% [6.8]	5.24 ± 0.67% [6.0]	4.53 ± 2.03% [5.2]
Height (mm)	20.05 ± 0.13% [20.0]	15.23 ± 0.14% [15.2]	10.42 ± 0.13% [10.4]	5.66 ± 0.43% [5.6]	20.02 ± 0.08% [20.0]	20.04 ± 0.09% [20.0]	20.07 ± 0.13% [20.0]	14.25 ± 0.12% [14.2]	11.33 ± 0.21% [11.3]	9.64 ± 0.20% [9.6]
Total Drug Dose (mg)	53.9 ± 6.5%	41.7 ± 2.1%	27.3 ± 1.4%	14.1 ± 4.4%	30.1 ± 11.9%	74.9 ± 2.5%	96.6 ± 6.2%	56.1 ± 1.0%	55.6 ± 1.1%	50.8 ± 2.0%
Relative Drug Content (%)	10.33 ± 4.92%	10.45 ± 0.67%	10.46 ± 1.10%	10.65 ± 0.29%	9.98 ± 5.64%	10.21 ± 0.45%	10.39 ± 0.95%	10.34 ± 1.09%	10.51 ± 0.22%	10.51 ± 1.45%

^1^ Identical implants of standard dimensions (also named A1, B2 and C1).

**Table 8:** Fitting quality of different kinetic equations (linearization plots) with mean release data points (<80%) from hollow cylindrical implants.

Implant Design		^1^ S	A2	A3	A4	B1	B3	B4	C2	C3	C4
Zero Order	*R*²	0.9739	0.9756	0.9766	0.9746	0.9742	0.9778	0.9805	0.9798	0.9803	0.9763
First Order	*R*²	0.9977	0.9980	0.9982	0.9973	0.9982	0.9970	0.9946	0.9958	0.9961	0.9974
Higuchi	*R*²	0.9963	0.9958	0.9955	0.9961	0.9980	0.9937	0.9923	0.9924	0.9926	0.9945
Weibull	*R*²	0.9907	0.9919	0.9918	0.9910	0.9884	0.9897	0.9874	0.9887	0.9898	0.9906
Korsmeyer-Peppas	*R*²	0.9997	0.9998	0.9996	0.9998	0.9995	0.9995	0.9992	0.9994	0.9995	0.9997
	*n*	0.592	0.600	0.590	0.600	0.550	0.600	0.602	0.611	0.609	0.607
	*log(k)*	1.59	1.57	1.56	1.59	1.87	1.45	1.38	1.45	1.36	1.28

^1^ Identical implants of standard dimensions (also named A1, B2 and C1).

**Table 9:** Characteristics of 3D printed implants with patient-specific shapes regarding their mass (mean ± RSD, *n* = 8), as well as total drug dose and relative drug content (mean ± RSD, *n* = 3). The relative drug content defines the mass of paracetamol related to the mass of the drug-loaded part of the implant (RSRL(80:20)_5T).

Implant Design	H1	H2	H3
Mass (mg)	885.0 ± 1.3%	433.3 ± 3.1%	1163.6 ± 2.2%
Total Drug Dose (mg)	57.0 ± 3.7%	25.7 ± 4.1%	71.0 ± 6.7%
Relative Drug Content (%)	10.29 ± 4.42%	9.95 ± 1.60%	9.76 ± 2.98%

## Data Availability

The data presented in this study are available upon request from the corresponding author.
